# Voluntary wheel running activates Akt/AMPK/eNOS signaling cascades without improving profound endothelial dysfunction in mice deficient in α-galactosidase A

**DOI:** 10.1371/journal.pone.0217214

**Published:** 2019-05-23

**Authors:** Justin J. Kang, Taylour A. Treadwell, Peter F. Bodary, James A. Shayman

**Affiliations:** 1 School of Kinesiology, University of Michigan, Ann Arbor, MI, United States of America; 2 Department of Pharmaceutical Sciences, College of Pharmacy, University of Michigan, Ann Arbor, MI, United States of America; 3 Division of Nephrology, Department of Internal Medicine, University of Michigan Medical Center, Ann Arbor, MI, United States of America; Medical College of Georgia at Augusta University, UNITED STATES

## Abstract

Fabry disease is caused by loss of activity of the lysosomal hydrolase α-galactosidase A (GLA). Premature life-threatening complications in Fabry patients arise from cardiovascular disease, including stroke and myocardial infarction. Exercise training has been shown to improve endothelial dysfunction in various settings including coronary artery disease. However, the effects of exercise training on endothelial dysfunction in Fabry disease have not been investigated. *Gla* knockout mice were single-housed in a cage equipped with a voluntary wheel (EX) or no wheel (SED) for 12 weeks. Exercised mice ran 10 km/day on average during the voluntary running intervention (VR) period. Despite significantly higher food intake in EX than SED, body weights of EX and SED remained stable during the VR period. After the completion of VR, citrate synthase activity in gastrocnemius muscle was significantly higher in EX than SED. VR resulted in greater phosphorylation of Akt (S473) and AMPK (T172) in the aorta of EX compared to SED measured by western blot. Furthermore, VR significantly enhanced eNOS protein expression and phosphorylation at S1177 by 20% and 50% in the aorta of EX when compared with SED. Similarly, plasma nitrate and nitrite levels were 77% higher in EX than SED. In contrast, measures of anti- and pro-oxidative enzymes (superoxide dismutase and p67phox subunit of NADPH oxidase) and overall oxidative stress (plasma oxidized glutathione) were not different between groups. Although the aortic endothelial relaxation to acetylcholine was slightly increased in EX, it did not reach statistical significance. This study provides the first evidence that VR improves Akt/AMPK/eNOS signaling cascades, but not endothelial function in the aorta of aged Gla deficient mice.

## Introduction

Fabry disease is an X-linked lysosomal storage disorder that results from a defective or absent activity of α-galactosidase A (GLA) [1]. The enzymatic defect leads to a progressive accumulation of glycosphingolipids including globotriaosylceramide, galabiosylceramide, and globotriaosylsphingosine. Glycosphingolipid accumulation is observed in a variety of cell types, notably in the endothelium and smooth muscle cells [2, 3]. Early symptoms of Fabry disease in childhood include episodic acute pain and gastrointestinal involvement with abdominal pain, diarrhea, and nausea [3]. However, premature life-threatening complications arise from cardiovascular diseases around the fourth decade of life, and include cerebrovascular events, myocardial infarction, hypertrophic cardiomyopathy, and renal failure [4]. Whereas enzyme replacement therapy (ERT) with recombinant GLA is a long standing approved treatment for Fabry disease, there is only limited evidence that ERT alters the natural course of cardiovascular morbidities in patients with advanced Fabry disease [5].

A mouse model of Fabry disease has been used to explore the vascular pathophysiology in the setting of GLA deficiency. Several inducible models of vasculopathy in these mice have demonstrated accelerated atherosclerosis, oxidant-induced occlusive arterial thrombosis, impaired acetylcholine-induced vascular relaxation, and the presence of endothelial nitric oxide synthase (eNOS) uncoupling [6–9]. A common basis for these experimentally observed abnormalities may be decreased nitric oxide (NO) bioavailability. Exercise has been shown to be one of the most effective non-pharmacological interventions for improving NO bioavailability [10]. During the last two decades, the beneficial effects of exercise on the vascular endothelium have been extensively studied in various aspects including endothelium-dependent vasodilation, anti-inflammation, and anti-atherosclerosis [11–13]. Furthermore, exercise has been demonstrated to improve acetylcholine-mediated coronary blood flow even in the setting of documented coronary artery disease [14, 15], suggesting that the presence of advanced disease does not preclude improvements in endothelial function resulting from exercise. Although exercise intolerance has been reported in patients with Fabry disease previously [16], a more recent study showed that exercise training could improve exercise capacity and well-being of Fabry patients who refrained from physical activity in the past [17]. This suggests that exercise training might be an alternative therapeutic option in Fabry disease. Yet, the benefits of exercise training with respect to vasculopathy in Fabry disease remain unclear.

The purpose of the present study, therefore, was to assess directly the effects of 12 weeks of voluntary wheel exercise training on signaling and functional alterations in the aorta using an established mouse model of Fabry disease. Specifically, we focused on aortic endothelial function, selected enzymes influencing the NO bioavailability in the endothelium (AMPK, Akt, eNOS expression and S1177, superoxide dismutase, and the p67phox subunit of NADPH oxidase), indirect markers of vascular oxidative/nitrosative stress level (3-nitrotyrosine), plasma markers of nitric oxide (nitrate and nitrite), and overall oxidative stress (oxidized glutathione).

## Materials and methods

### Mice

*Gla* null mice (129/SvJXC57BL/6J) used in this study were bred from mice originally generated and provided by Drs. Ashok Kulkarni and Roscoe Brady (National Institute of Health, Bethesda, MD) as described previously [18]. These mice were back-crossed a minimum of six generations to the C57BL/6J strain. The animals were maintained on a 12-h light/dark cycle with free access to food and water ad libitum. A week before the voluntary wheel intervention, groups of male mice (8–11 months) with similar average body weight were single-housed and assigned to either a control sedentary (n = 20, SED) or voluntary wheel running group (n = 20, EX). Mice in the EX group were provided with a running wheel (5” diameter x 2” width) equipped with an odometer (Bell dashboard 100-F12) for 12 weeks. Running distance and food intake were monitored daily. Body weight was measured weekly. All animal experiments were conducted according to the protocol, which was approved by the Institutional Animal Care and Use Committee of the University of Michigan.

### Tissue harvest

Mice were euthanized with an injection of pentobarbital sodium (66.5 mg/kg ip) at approximately 0900 h. Due to the logistics of the functional studies of the aorta, one mouse from each group was euthanized per day on consecutive days at the end of the 12-week intervention. The remaining mice not used in the functional studies were euthanized at the 12-week intervention time point. Animals in the EX group had access to the voluntary wheel up until the time of tissue collection.

### Aortic protein expression

The thoracic aorta was dissected, cleared of surrounding connective tissues in cold physiological salt solution (PSS, mmol/L: 130 NaCl, 4.7 KCl, 1.18 KHPO_4_, 1.17 MgSO_4_, 1.6 CaCl_2_, 14.9 NaHCO_3_, 5.5 dextrose, and 0.03 EDTA), and frozen in liquid nitrogen. The vessels were pulverized in liquid nitrogen using a pestle. Each powdered sample was lysed with 200 μl RIPA lysis buffer (#R0278, Sigma) with 1X mixture of phosphatase/protease inhibitors (#P2714, P0044, and P5726, Sigma). Homogenates were then incubated on a rotor at 4°C for 1 hr. Cell debris was removed by centrifugation at 10,000 x g for 10 min at 4°C. Quantification of total protein in each aortic lysate sample was determined by a bicinchoninic acid assay with bovine serum albumin as a standard. For measures of aortic protein expression, 15 μg of protein with Laemmli sample buffer were loaded on 4–12% gradient gels, separated by electrophoresis, and transferred onto a nitrocellulose membrane. The membrane was blocked in 5% non-fat dry milk in Tris-buffered saline with 0.1% Tween-20 (TBST) for at least 1 hr at room temperature. After blocking, the membrane was washed with TBST and incubated with primary antibody overnight at 4°C followed by washing with TBST. Primary antibodies used were eNOS (#ab76198, Abcam), phospho-eNOS Ser^1177^ (#9571, Cell Signaling), Akt (#2938, Cell Signaling), phospho-Akt Ser^473^ (#4060, Cell Signaling), AMPK (#5831, Cell Signaling), phospho-AMPK Thr^172^ (#2531, Cell Signaling), 3-nitrotyrosine (#ab7048, Abcam), VASP (#3112, Cell Signaling), phospho-VASP Ser^239^ (#3114, Cell Signaling), SOD1(#ab13498, Abcam), SOD2 (#ab16956, Abcam), SOD3 (#S4946, Sigma), and p67phox (#610912, BD Bioscience). The membrane was incubated with appropriate secondary antibody. The immunoreactive bands were detected with ECL western blotting substrate (#32106, Thermo Fisher), and quantified by densitometric scanning using ImageJ software.

### Citrate synthase activity assay

Gastrocnemius muscles from SED and EX mice were dissected and flash frozen in liquid nitrogen. Frozen muscles were weighed, transferred to pre-chilled glass tissue grinding tubes (Kontes, Vineland, NJ), and homogenized 1:20 (wt:vol) in ice-cold lysis buffer (50mM TRIS-HCl, 1mM EDTA, 0.1% Triton X-100, pH 7.2) using a glass pestle attached to a motorized homogenizer (Caframo, Wiarton, ON). Homogenates were then incubated on a rotor at 4°C for 1 hr. The lysates were centrifuged at 10,000 x g for 10 min at 4°C to remove cell debris. Quantification of total protein in each aortic lysate sample was performed by a bicinchoninic acid assay with bovine serum albumin as a standard. Two micrograms of gastrocnemius homogenate were used. Citrate synthase activity was determined using an assay kit according to the instructions (#701040, Cayman).

### Plasma nitric oxide level

Plasma nitric oxide levels were determined by photometric analysis using a nitrate/nitrite assay kit according to the instructions (#780001, Cayman). One hundred microliter of plasma was filtered through a 10 kDa molecular weight cut-off filter by centrifugation at 14,000 x g for 30 min at 4°C in order to reduce background absorbance due to the presence of hemoglobin.

### Glutathione measurements

The level of oxidized glutathione (GSSG) was determined using a commercially available glutathione (GSSG/GSH) assay kit (#STA-312, Cell Biolabs). Total glutathione level (oxidized + reduced glutathione) was determined by adding glutathione reductase. GSH level was determined without the enzyme.

### Assessment of aortic endothelial function

Following tissue harvesting, the thoracic aortae were dissected and placed in a dissection Petri dish filled with cold PSS. After removing connective tissue, segments (2–3 mm in length) of aorta were mounted on pins in a myograph system (model 610M, Danish Myo Technology, Aarhus, Denmark). Vessels were slowly warmed (37°C) and aerated (95% O_2_ and 5% CO_2_) in PSS for 20 min. Rings were set at 700 mg passive tension and equilibrated for 60 min with washing with pre-warmed and aerated PSS every 20 min. Prior to performing concentration response curves, vessels were subjected to osmotically balanced 60 mM potassium physiological salt solution (KPSS, mmol/l: 14.7 NaCl, 100 KCl, 1.18 KHPO_4_, 1.17 MgSO_4_, 1.6 CaCl_2_, 14.9 NaHCO_3_, 5.5 dextrose, and 0.03 EDTA). After washing, the vessels were contracted with 100 mmol/L KPSS until a plateau was attained, followed by washes. Phenylephrine (PE, 10^−9^ mol/L to 10^−4^ mol/L) was added cumulatively to establish a concentration-response curve, in which PE EC_80_ was calculated for each individual ring. The vessels were contracted with their individual PE EC_80_ values and allowed to reach a stable plateau. Subsequently, acetylcholine (ACh) or sodium nitroprusside (SNP) was added cumulatively to the chamber to determine endothelium-dependent (ACh) or endothelium-independent (SNP) relaxation. All chemicals used in the vascular reactivity study were purchased from Sigma (St. Louis, MO). The vascular reactivity to ACh or SNP was presented on a percent basis according to the following formula: Relaxation (%) = (T_PE80_ –T_d_) / (T_PE80_) x 100, where T_PE80_ is the steady-state tension produced after addition of PE EC80, and T_d_ is the steady state tension following addition of vasoactive agent (ACh, SNP). Sensitivity (EC_50_) was defined as the concentration of the agent that produced 50% of its maximal response. A total of eight mice per group were utilized for these studies.

### Statistical analysis

GraphPad Prism software was used for statistical analysis. The data were presented as mean ± standard error of the mean. Results were analyzed using the unpaired t-test for comparison of two groups. For vascular reactivity studies using ACh or SNP, concentration-response data was analyzed using two-way ANOVA (exercise and concentration) to compare the concentration-response curves between the EX and SED groups. Bonferroni’s post hoc test was used to assess differences at individual points on the concentration-response curves if the results of the two-way ANOVA comparison between curves were significantly different. Statistical significance was set at p<0.05.

## Results

### Overall effects of voluntary wheel running on non-vascular endpoints

**Fig 1:**
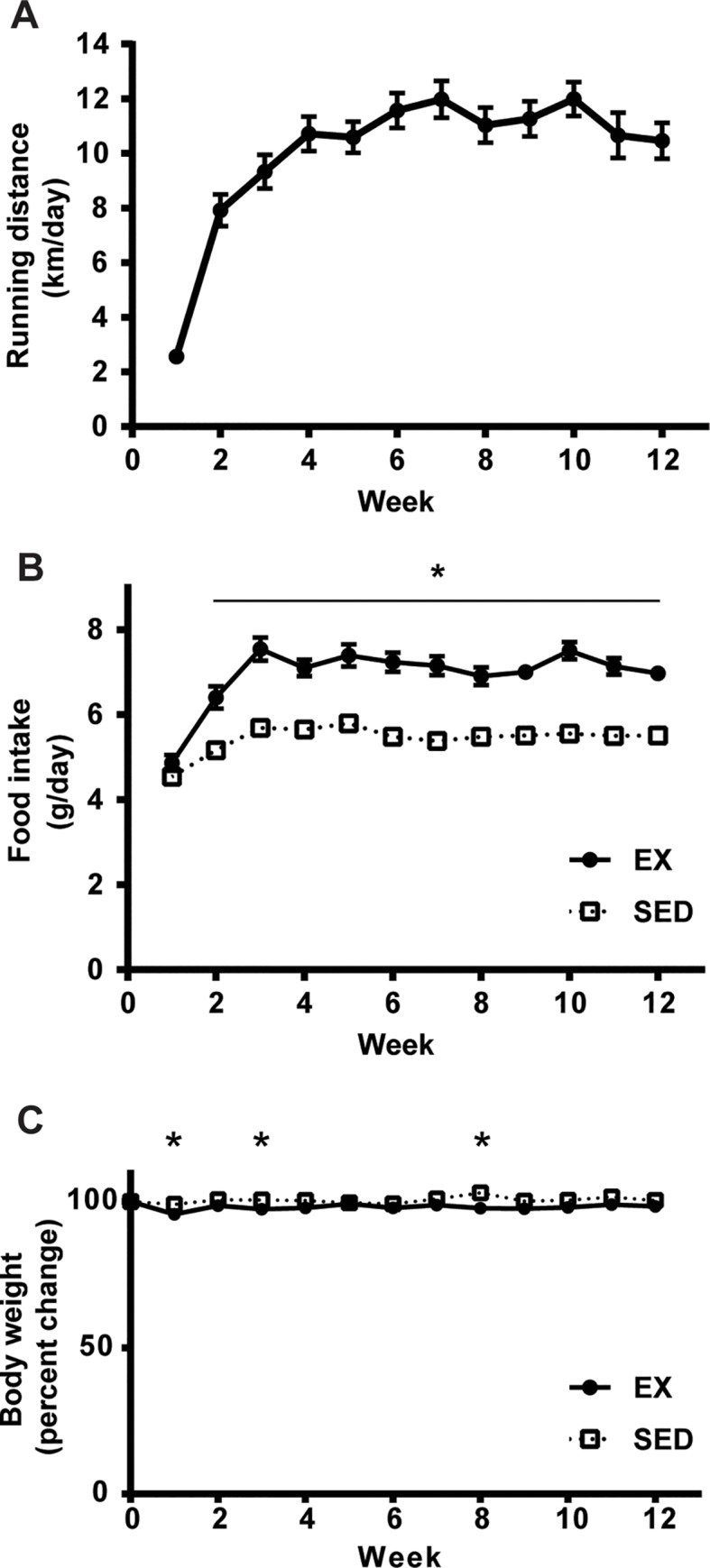
Daily running distance and changes in food intake and body weight in EX and SED mice during 12 weeks of voluntary wheel intervention. Graphs show **A.** the average daily running distance (km/day), **B.** food intake (g/day); *p<0.001 compared to SED mice, and **C.** changes in body weight expressed as percentage of that at the beginning of the 12-week voluntary wheel intervention; *<0.05 compared to SED mice (n = 19 per group). The data represent the mean ± SEM.

**Fig 2:**
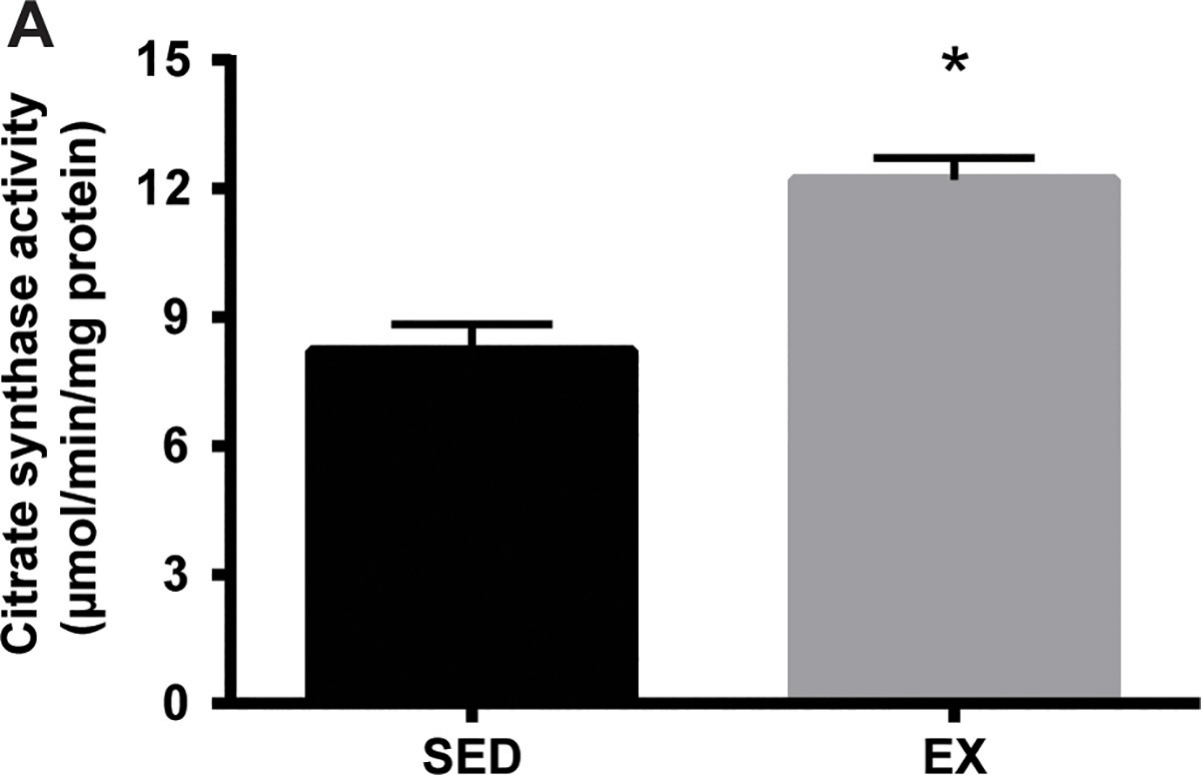
Increased citrate synthase activity in gastrocnemius muscle from EX mice. Gastrocnemius muscle was homogenized as described in the methods section. Citrate synthase activity in muscle homogenate was determined by measuring the production of SH-CoA from the condensation of dicarboxylate oxaloacetate and acetyl CoA by citrate synthase (n = 8 per group). The data are expressed as μmol/min/mg protein. *p < 0.001 compared to SED mice.

The mice in the voluntary running wheel group (EX) ran on average 10 ± 0.75 km/day (Fig 1A). During the first week of the exercise intervention, food intake was not different between the mice in the sedentary control (SED) and EX groups (Fig 1B). In the second week, consistent with the increased running distance, EX mice consumed 24% more food compared to SED mice. Interestingly, body weights were not different at the completion of voluntary wheel running (VR) compared to the beginning of VR in both SED and EX mice (Fig 1C). Despite no changes in total body mass, 12 weeks of VR resulted in a significantly higher heart to body mass ratio and lower gonadal fat to body mass ratio in EX mice compared to those in SED mice (Table 1). The activity of citrate synthase as a marker for mitochondrial content was also measured to confirm an exercise training effect [19, 20]. Consistent with the results in running distance, there was a 49% increase in the citrate synthase activity in gastrocnemius muscle of EX mice compared with SED controls (Fig 2).

**Table 1:** Summary of parameters of SED and EX mice.

	SED	EX
Final body weight (g)	35.1 ± 0.6	34.0 ± 0.6
Food intake (g/day)	5.4 ± 0.1	6.9 ± 0.2*
Heart (mg)	198.4 ± 5.6	225.0 ± 6.2*
Heart:BW (g/g x 100)	0.57 ± 0.01	0.66 ± 0.01*
Gonadal fat (mg)	533.7 ± 46.1	277.9 ± 18.7*
GF:BW (g/g x 100)	1.50 ± 0.12	0.82 ± 0.06*

Data are shown as mean ± SEM (n = 19 per group).

*p < 0.01 compared to SED mice.

### VR increases Akt / AMPK / eNOS signaling pathways in the aorta of EX mice

**Fig 3:**
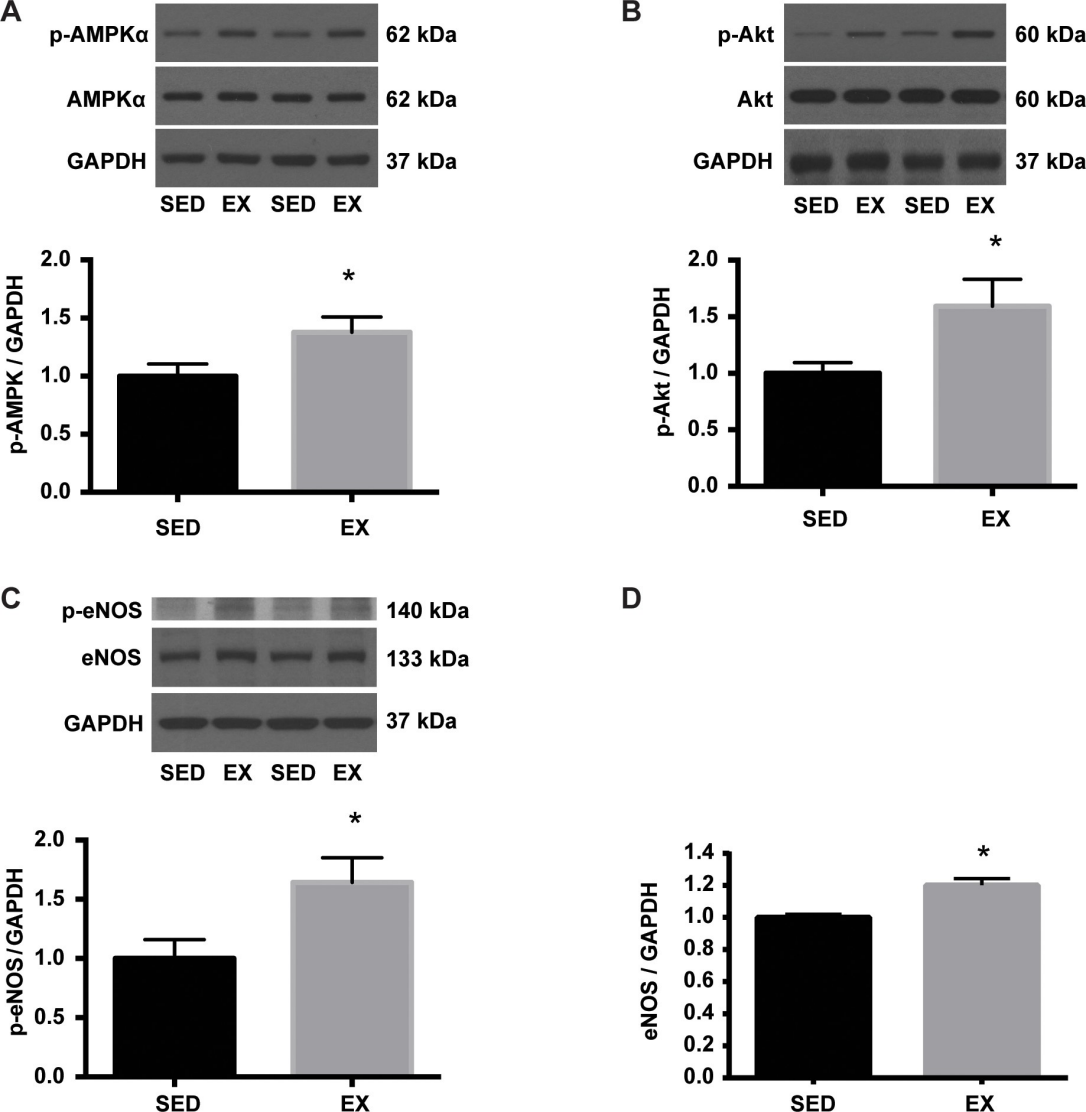
Increased p-AMPK, p-Akt, and p-eNOS in the aorta of EX mice. Equal amounts of aortic homogenate were separated by SDS-PAGE. Representative blots are shown above each summary graph. **A.** Phospho-AMPKα (Thr172) was normalized to GAPDH expression and expressed relative to SED mean (n = 7 per group). *p<0.05. **B.** Phospho-Akt (Ser473) was normalized to GAPDH expression and expressed relative to SED mean (n = 7 per group). *p<0.05. **C.** Phospho-eNOS (Ser1177) was normalized to GAPDH expression and expressed relative to SED mean (n = 7 per group). *p<0.01. **D.** Total eNOS protein expression was normalized to GAPDH and expressed relative to SED mean (n = 7 per group). *p<0.001.

Exercise has been shown to increase eNOS activity, in part, through shear stress [21]. Akt and AMPK have been proposed to be critical mediators of shear stress-induced eNOS activation [22]. The expression and phosphorylation status of Akt, AMPK, and eNOS were determined to test the hypothesis that VR induced changes in signaling pathways in the aorta. The level of p-AMPK (Thr-172) normalized to GAPDH (arbitrary units; 100 ± 10% vs. 138 ± 13%: SED vs. EX) as well as the ratio of p-AMPK (Thr-172) / t-AMPK (arbitrary units; 100 ± 9% vs. 137 ± 13%: SED vs. EX) were significantly higher in the aorta of EX than SED mice (Fig 3A). A significant increase in the p-Akt (Ser-473) / GAPDH (arbitrary units; 100 ± 25% vs. 159 ± 24%: SED vs. EX) and p-Akt (Ser-473) / t-Akt ratio (arbitrary units; 100 ± 9% vs. 148 ± 17%: SED vs. EX) were also noted in the aorta of EX mice in comparison with SED mice (Fig 3B). It has been shown that phosphorylation of eNOS at Ser1177 is a major downstream target of Akt and AMPK in response to exercise and shear stress [21]. Consistent with the previous finding, we observed a significantly higher levels of p-eNOS (Ser-1177) to GAPDH (arbitrary units; 100 ± 16% vs. 164 ± 21%: SED vs. EX) and p-eNOS (Ser-1177) to total eNOS ratio (arbitrary units; 100 ± 10% vs. 154 ± 12%: SED vs. EX) in the aorta of EX mice when compared to that of SED mice (Fig 3C). In addition, the ratio of eNOS to GAPDH protein expression was significantly elevated (arbitrary units; 100 ± 2% vs. 120 ± 4%: SED vs. EX) in the aorta of EX in comparison with SED mice (Fig 3D). These results suggested that 12 weeks of VR intervention mediated an increase in expression and activity of eNOS and its upstream signaling kinases.

### Nitric oxide and oxidative stress level

**Fig 4:**
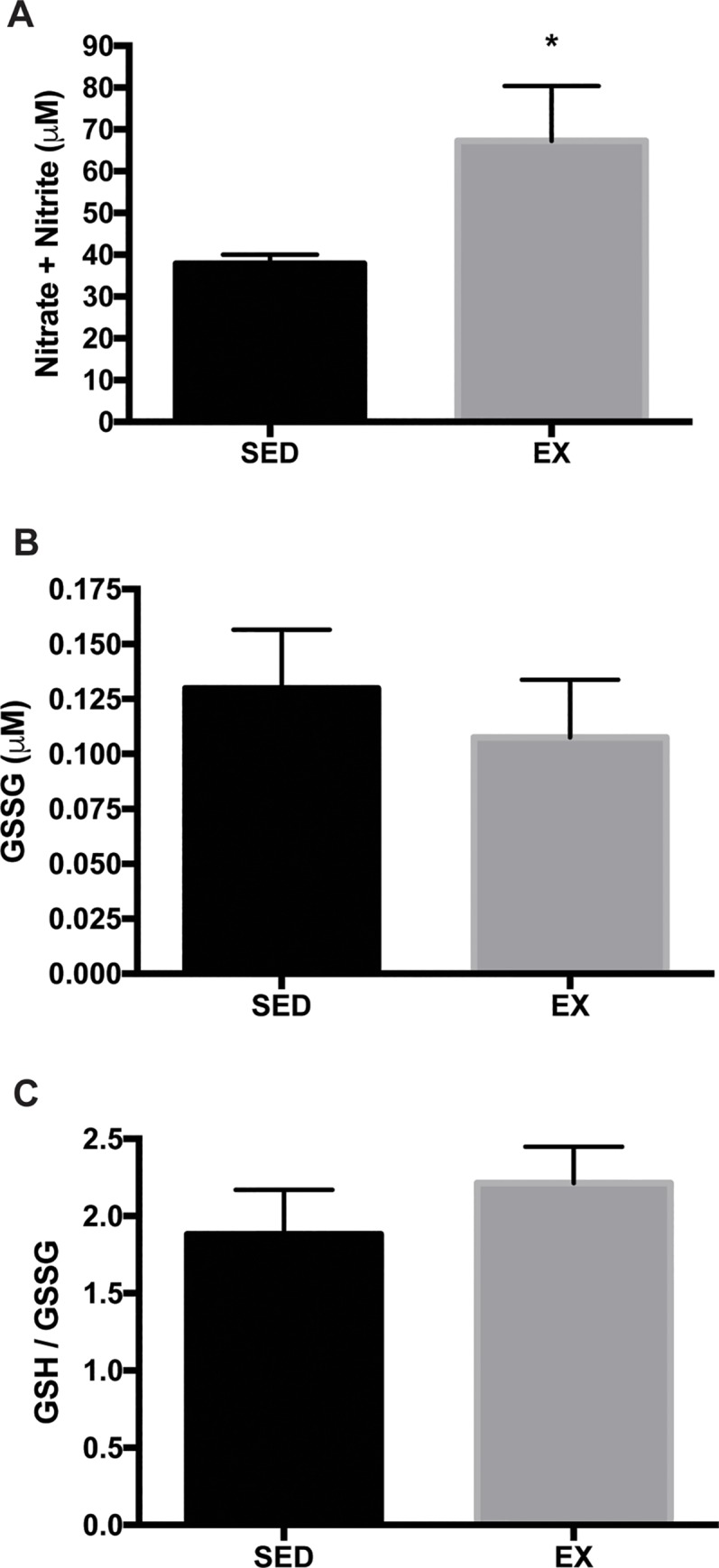
Levels of plasma NO and oxidized glutathione. **A.** Hemoglobin-free plasma was used to determine total nitrate and nitrite levels (n = 8 per group). *p<0.05. **B.** Metaphosphoric acid (5%)-treated plasma was used to determine oxidized glutathione levels and GSH/GSSG ratio (n = 17 per group).

**Fig 5:**
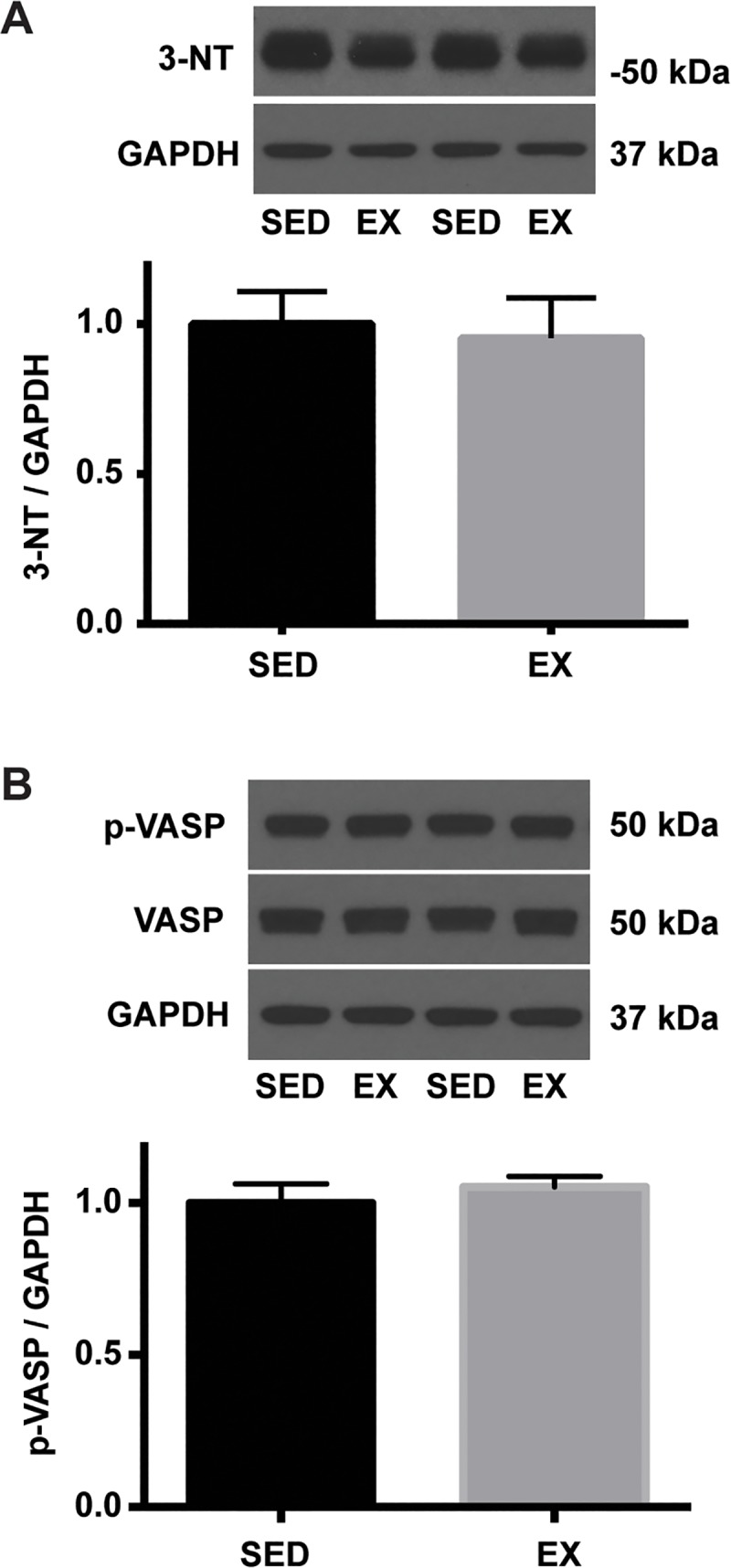
Levels of 3-nitrotyrosine and NO bioavailability in the aortic tissue of SED and EX mice. An equal amount of aortic homogenates was separated by SDS-PAGE. Representative blots are shown above each summary graph. **A.** 3-nitrotyrosine abundance was normalized to GAPDH and expressed relative to SED mean (n = 7 per group). **B.** Phospho-VASP (Ser239) level was normalized to GAPDH expression and expressed relative to SED mean (n = 7 per group).

**Fig 6:**
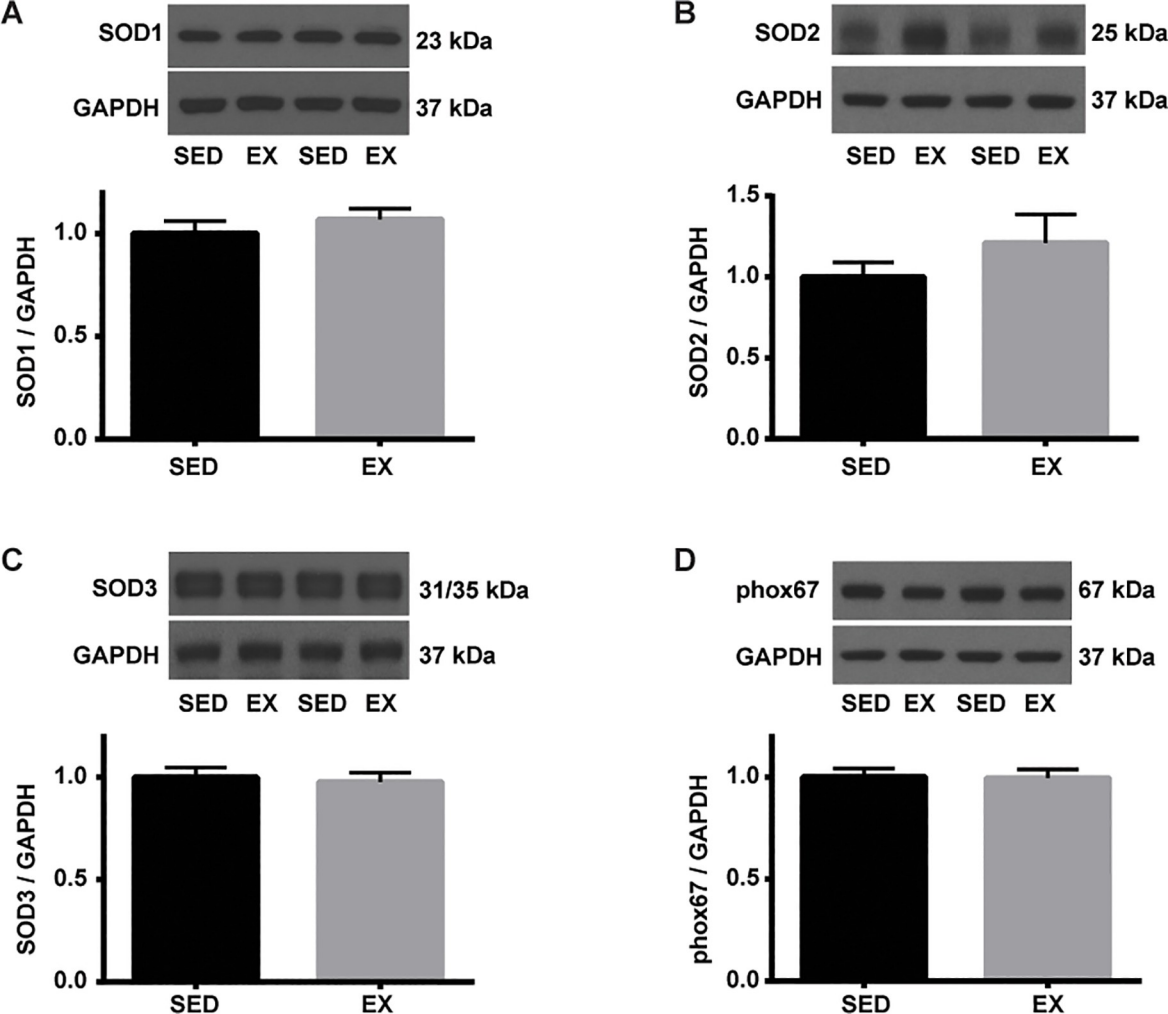
Levels of SOD and p67phox subunit of NADPH oxidase in the aorta of SED and EX mice. An equal amount of aortic homogenates was separated by SDS-PAGE. Representative blots are shown above each summary graph. Each protein level was normalized to GAPDH and expressed relative to SED mean (n = 7 per group). **A.** Representative blots of SOD1. **B.** Representative blots of SOD2. **C.** Representative blots of SOD3. **D.** Representative blots of p67phox, a subunit of NADPH oxidase.

We previously reported that GLA deficiency results in eNOS uncoupling and reduced NO bioavailability [23]. Citrated plasma was used to determine the effects of VR on NO and oxidative stress levels. VR resulted in a significantly higher level of NO measured by total nitrate and nitrite levels (37.8 ± 2.2 μM vs. 67.2 ± 13.1 μM: SED vs. EX) (Fig 4A), which was in line with the increased total and phospho/total eNOS levels in EX than SED mice. However, the overall oxidative stress level, determined by measurements of GSSG level (0.13 ± 0.03 μM vs. 0.11 ± 0.03 μM: SED vs. EX) as well as GSH/GSSG ratio (arbitrary unit; 1.88 ± 0.29 vs. 2.21 ± 0.24) were not different between groups following 12 weeks of VR (Fig 4B). We and others have observed increased levels of oxidative/nitrosative stress in tissues of patients with Fabry disease and GLA-deficient mice [24–26]. Thoracic aortae were used to determine the effects of VR on 3-nitrotyrosine (NT) abundance, a cellular marker of peroxynitrite formation, therefore vascular oxidative/nitrosative stress [27]. In this study, VR did not alter the level of NT in the aortic tissue (Fig 5A), suggesting the balance between NO and superoxide did not change significantly by VR. NO level in response to exercise was next determined in the aorta. Soluble guanylate cyclase is the major physiological receptor for NO and catalyzes the synthesis of intracellular cGMP level [28]. Protein kinase G is activated by cGMP and preferentially phosphorylates vasodilator-stimulated phosphoprotein (VASP) at Ser-239 [29, 30]. Therefore, VASP is a common marker used for monitoring NO-mediated signaling [30]. However, we observed no changes in both p-VASP (Ser 239) to GAPDH and p-VASP (Ser 239) to t-VASP ratio in response to 12 weeks of VR (Fig 5B). In addition, VR had no effect on the protein expression of Mn-, CuZn-, and ec-SOD in the aortae of EX compared to SED mice (Fig 6A–6C). Finally, we determined the effect of VR on NADPH oxidase, a prominent source of vascular-derived reactive oxygen species, in the aorta [31]. p67phox is one of the subunits of NADPH oxidase, and has been found to be reduced in aorta of mice following VR training [12]. In the present study, VR did not alter p67phox subunit protein expression in EX mice compared to SED (Fig 6D).

### Endothelial function

**Fig 7:**
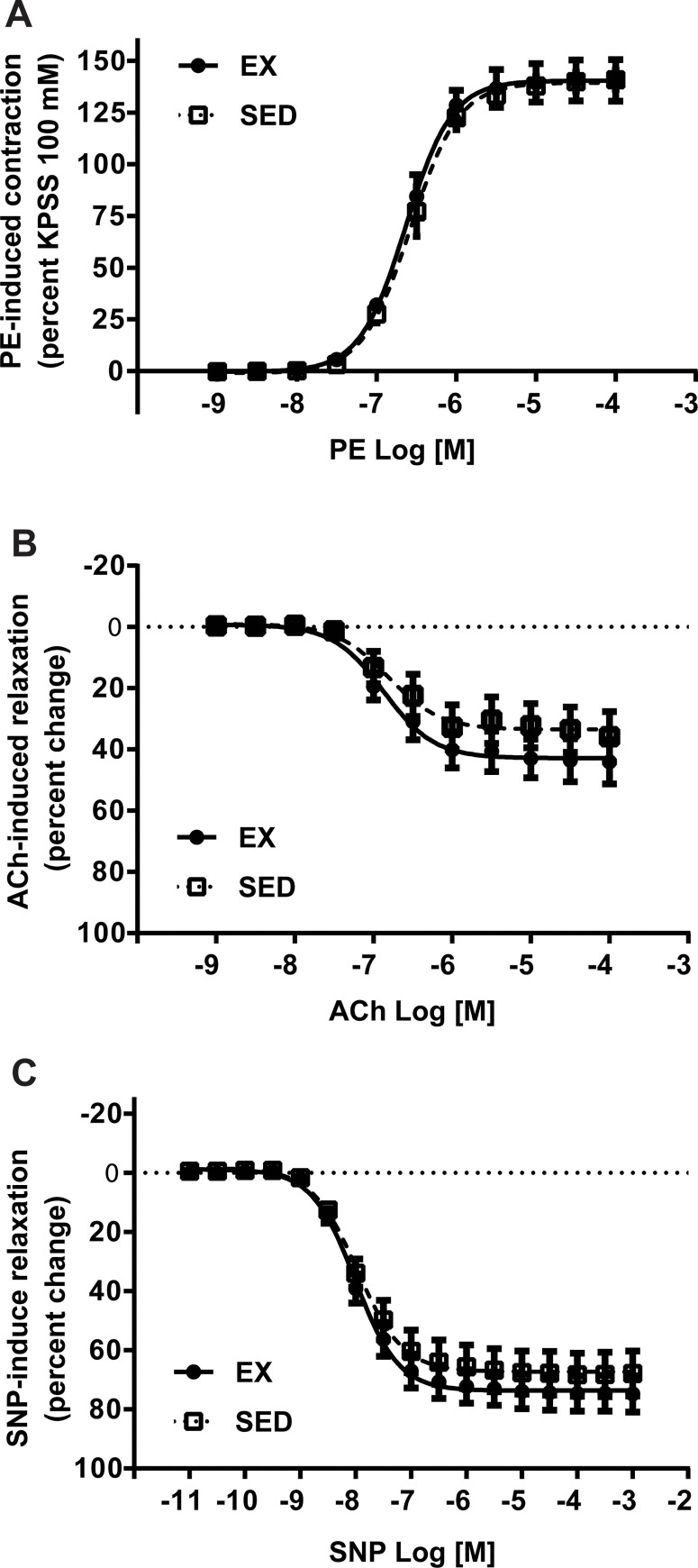
Endothelium-dependent and -independent aortic vascular relaxation in SED and EX mice. **A.** Phenylephrine (PE)-mediated vascular contraction in aortic rings from SED and EX mice was expressed as a percentage maximum response to KPSS (n = 8 per group). **B.** Acetylcholine (ACh)-mediated endothelium-dependent relaxation in the aortic rings from SED and EX mice was expressed as a percentage relaxation of the pre-contraction elicited by PE EC_80_ (n = 8 per group). **C.** Sodium nitroprusside (SNP)-mediated endothelium-independent relaxation in the aortic rings from SED and EX mice was expressed as a percentage relaxation of the pre-contraction elicited by PE EC_80_ (n = 8 per group).

The vascular contraction mediated by 100 mmol/L KPSS was equivalent in aortae from SED and EX mice (1351.81 ± 117.09 mg vs. 1511.36 ± 105.54 mg, n = 8/group, p>0.05). Phenylephrine (PE) caused a concentration-dependent contraction in isolated aortic rings from both SED and EX mice (Fig 7A). The PE-induced contractions were equivalent in aortae from SED and EX mice as demonstrated by similar log EC_50_ values (-6.58 ± 0.01 vs. -6.63 ± 0.02, p>0.05) as well as equivalent E_max_ values (140.83 ± 4.71% vs. 140.60 ± 10.01%, p>0.05). Based on the PE-induced contraction response, PE EC_80_ was calculated for each aortic ring. Receptor-mediated endothelium-dependent relaxation to acetylcholine (ACh) was examined in aortic rings from SED and EX mice. The vessels from SED and EX mice were relaxed in response to ACh in a concentration-dependent manner (Fig 7B). Both the maximal relaxation elicited by ACh (36.21 ± 7.88% vs. 44.0 ± 7.04%, p>0.05) and log EC_50_ values (-6.73 ± 0.14 vs. -6.78 ± 0.12, p>0.05) did not differ between the groups. Sodium nitroprusside (SNP) induced a concentration-dependent, endothelium-independent relaxation in isolated aortae from SED and EX mice (Fig 7C). SNP-mediated vasodilation was greater in the aortic rings from both SED and EX mice (67.89 ± 7.48% vs. 74.75 ± 6.05%) than the endothelium-dependent dilation to ACh (Fig 7C), suggesting the low magnitude of relaxation to ACh was not due to changes in the sensitivity of vascular smooth muscle to NO.

## Discussion

Previous research regarding Fabry disease has focused on the pathophysiologic mechanisms using both patients and mouse models [6–8, 32–34]. However, few studies have investigated the potential effects of exercise training on endothelial dysfunction in Fabry disease [17]. In this study, we examined whether 12 weeks of VR intervention could improve endothelial dysfunction in the presence of eNOS uncoupling in a mouse model of Fabry disease. Our results indicate that in aged mice with Fabry disease, VR (a) induced exercise training adaptations, (b) increased Akt/AMPK/eNOS signaling pathways in the aorta, (c) increased plasma NO levels, but (d) did not improve endothelial dysfunction and systemic markers of oxidative stress in the aorta.

Our finding that VR induced training adaptations in tissues was supported by an increase in heart size and skeletal muscle citrate synthase activity. After 12 weeks of VR, a heart to total body mass ratio was higher in EX compared to SED mice. In addition, the magnitude of the increase in citrate synthase activity observed in EX compared to SED mice was comparable with a previous study examining the skeletal muscle of wild-type C57BL/6J mice following voluntary wheel exercise [35].

EX mice had 54% and 20% higher levels of aortic Ser1177 phosphorylation and protein expression of eNOS, respectively, compared to SED mice. One possible mechanism by which VR augmented eNOS activity as well as protein expression is through an activation of upstream kinases of eNOS by an increase in shear stress during exercise. Several lines of evidence have demonstrated that eNOS mRNA and protein expression are increased in endothelial cells exposed to shear stress [36, 37], in isolated coronary arterioles subjected to elevated intraluminal flow [38, 39], and in the aorta from exercise trained mice [37, 40–42] and rats [43]. In a previous study, shear stress increased eNOS activity, measured by NO production and phosphorylation of eNOS at S1177, via PI(3)K/Akt-dependent pathway in HUVEC cells, which was prevented by wortmannin and in cells transfected with dominant-negative Akt mutant [44]. Zhang et al. showed that arterial p-eNOS S617, which is activated by Akt alone, and p-eNOS S1177, which is activated by both Akt and AMPK, were increased in response to treadmill-running in mice [21]. In the same study, intraperitoneal administration of wortmannin before treadmill running revealed either Akt or AMPK alone might be sufficient to activate p-eNOS S1177 during exercise. As such, we determined activation status of Akt and AMPK in the aorta in response to VR in this study. In keeping with the previous findings, we observed significantly elevated levels of phospho -Akt (S473), -AMPK (T172), and -eNOS (S1177) in the aorta as well as higher plasma NO level in EX as compared to SED mice.

We have previously published that endothelial dysfunction in the aorta is evident in 3–5 month-old GLA deficient compared to WT mice, characterized by decreased maximal vasodilation to ACh in the aorta (E_max_: ~60%) [8]. In the present study, the E_max_ to ACh in GLA deficient mice at 11–13 months was approximately 40%. Another group reported 25% of E_max_ to ACh in the same mice at 19 months [45]. These results are consistent with a progressive decline in endothelial function in Fabry disease. Indeed, endothelial dysfunction in the mesenteric artery and accelerated oxidant-induced thrombosis in the setting of GLA deficiency in other studies were all shown to be age-dependent [7, 9]. A previous study by Durrant el al. showed that carotid artery vasodilatation to ACh was improved in older mice of an aging model subjected to 10–14 weeks of voluntary running compared to their age-matched sedentary counterparts [12]. On the other hand, some studies have reported that exercise did not improve endothelial function and/or arterial stiffness despite significant improvement in VO_2_ peak or p-eNOS S1177 [46, 47].

In this study, despite the documented response to training adaptation, VR did not improve endothelial relaxation in the aged Fabry mice. One possible reason might be the rapid inactivation of NO by reactive oxygen species (ROS). Indeed, the dose-dependent Gb3 accumulation in cultured vascular endothelial cells resulted in increased intracellular ROS [34]. In the presence of elevated ROS, NO binds to superoxide to form peroxynitrite, a powerful oxidizing intermediate [48]. NT has been used as a specific marker of the presence of reactive oxygen/nitrogen species [12, 49]. In a previous study, an age-related increase in NT in the aorta of older mice of an aging model was attenuated by VR with a corresponding increase in SOD and decreased NADPH oxidase activity [12]. Although we observed that plasma NO was significantly increased, the overall pattern of oxidative stress levels did not change following VR in the current study. Furthermore, the ratio of phospho to total VASP and the expression of SODs and p67phox, an important regulatory subunit of NADPH oxidase, did not differ significantly between EX and SED mice, suggesting that there were no changes in the balance between NO availability and oxidative/nitrosative stress level.

A second potential explanation for the lack of benefit in VR may be the possible presence of other sources of oxidants. For example, H_2_O_2_ has been observed to induce protein kinase G dimerization and vasorelaxation, which was associated with phosphorylation of VASP at Ser^239^ [50]. In addition, we have previously observed a 6-fold higher level of aortic 3-nitrotyrosine in *Gla* null mice at 8 months compared with age-matched wild-type mice [25]. Moreover, inducible NOS expression was more than 2 fold higher in the aorta obtained from 12-month-old *Gla* deficient mice than their wild-type counterpart [6]. In a recent study, Silva et al. showed that 8 weeks of treadmill exercise training resulted in attenuated iNOS and increased phospho- and total eNOS expressions in the mouse aorta [51]. Therefore, it is possible that other sources of oxidants may have been altered following VR, resulting in no difference in the overall expression of NT as well as phosphorylation of VASP.

A third potential reason for the lack of improvement in endothelial function in the aorta might be advanced morphological alterations of the smooth muscle cells and extracellular matrix that preceded the VR intervention. The aortae from GLA deficient mice displayed less sensitive endothelium-independent relaxation with an NO donor compared to wild type mice, suggesting alterations in vascular smooth muscle cells (SMC) [8]. Heare et al. reported that aortic wall thickness of *Gla*-deficient mice was significantly increased with high Gb3 storage level in endothelium and vascular SMC compared with wild type mice [45]. Progressive thickening of the intima-media layers of radial arteries was also observed in patients with Fabry disease [52]. One hypothesis is that accumulation of lyso-Gb3 (globotriaosylsphingosine) in the SMC promotes SMC proliferation resulting in increased intima-media thickness in the setting of Fabry disease [53]. Gb3 accumulation in the endothelial cells has also been reported to increase ROS and cellular adhesion molecules [34]. Often, these pathological conditions are associated with inflammation, hypertrophy, apoptosis, and replacement fibrosis in older patients with Fabry disease [54, 55]. Fabry patients without myocardial fibrosis, compared to those with myocardial fibrosis, showed better outcomes regarding left ventricular mass, improved myocardial function, and a higher exercise capacity during 3 years of enzyme replacement therapy [56]. Thus, VR may not be able to reverse the age-related alterations that have accumulated before the initiation of the intervention in Fabry disease. Taken together, these data indicate that our exercise intervention does not reverse endothelial dysfunction and oxidative stress level in these aged GLA-deficient mice.

Several limitations exist in our study. First, age-matched wild-type mice were not included for a direct comparison of the measurements with GLA-deficient mice. However, we and others have previously demonstrated endothelial dysfunction in younger and older mice with Fabry disease compared to age-matched wild-type mice [8, 9, 45]. In addition, the primary goal of the present study was to gain initial insight into the effects of exercise on endothelial dysfunction and changes in signaling pathways in aged mice with Fabry disease. Finally, we assessed the levels of signaling kinases in aortic homogenates rather than in the aortic endothelial cells alone. However, a recent study, using phospho-protein-specific antibodies with immunohistochemistry analysis, demonstrated that exercise induced an activation of AMPK with concurrent elevation of p-eNOS S1177 in the endothelial cells to a much greater extent than those in the smooth muscle cells in the aorta of mice [57]. Although our study bears some limitations, this is the first study to evaluate the influence of voluntary running on endothelial function in a mouse model of Fabry disease.

In conclusion, VR significantly improved Akt/AMPK/eNOS signaling pathways without improvement of the severe endothelial dysfunction evident in the aorta of aged mice with Fabry disease. This finding may have relevance to the clinical studies of Fabry disease as well. Emerging clinical data evaluating the effectiveness of long-term enzyme replacement therapy indicated that this therapy does not prevent the occurrence of new cardiovascular complications in Fabry patients with more advanced disease [58]. Furthermore, another recent study demonstrated that Fabry patients with no detectable fibrosis and mild hypertrophy, compared to those with myocardial fibrosis, showed better outcomes regarding left ventricular mass, improved myocardial function, and a higher exercise capacity during 3 years of enzyme replacement therapy [56]. Therefore, evidence is accumulating that early intervention may have more beneficial effects on these patients. Similarly, the findings of this study raise a primary question of whether exercise might suppress the progression of endothelial dysfunction in this setting if started earlier in this disease process. Future studies examining the effects of exercise in younger age or as an adjuvant treatment with enzyme replacement therapy will further our understanding of the effects of exercise as a potential strategy for preventive and therapeutic interventions for vasculopathy in Fabry disease.
